# DNA barcoding and LC-MS metabolite profiling of the lichen-forming genus *Melanelia*: Specimen identification and discrimination focusing on Icelandic taxa

**DOI:** 10.1371/journal.pone.0178012

**Published:** 2017-05-24

**Authors:** Maonian Xu, Starri Heidmarsson, Margret Thorsteinsdottir, Finnur F. Eiriksson, Sesselja Omarsdottir, Elin S. Olafsdottir

**Affiliations:** 1 Faculty of Pharmaceutical Sciences, University of Iceland, Reykjavik, Iceland; 2 Akureyri Division, Icelandic Institute of Natural History, Akureyri, Iceland; 3 ArcticMass, Reykjavik, Iceland; National Cheng Kung University, TAIWAN

## Abstract

Taxa in the genus *Melanelia* (Parmeliaceae, Ascomycota) belong to a group of saxicolous lichens with brown to black foliose thalli, which have recently undergone extensive changes in circumscription. Taxa belonging to Parmeliaceae are prolific producers of bioactive compounds, which have also been traditionally used for chemotaxonomic purposes. However, the chemical diversity of the genus *Melanelia* and the use of chemical data for species discrimination in this genus are largely unexplored. In addition, identification based on morphological characters is challenging due to few taxonomically informative characters. Molecular identification methods, such as DNA barcoding, have rarely been applied to this genus. This study aimed to identify the *Melanelia* species from Iceland using DNA barcoding approach, and to explore their chemical diversity using chemical profiling. Chemometric tools were used to see if lichen metabolite profiles determined by LC-MS could be used for the identification of Icelandic *Melanelia* species. Barcoding using the fungal nuclear ribosomal internal transcribed spacer region (nrITS) successfully identified three *Melalenlia* species occurring in Iceland, together with *Montanelia disjuncta* (Basionym: *Melanelia disjuncta*). All species formed monophyletic clades in the neighbor-joining nrITS gene tree. However, high intraspecific genetic distance of *M*. *stygia* suggests the potential of unrecognized species lineages. Principal component analysis (PCA) of metabolite data gave a holistic overview showing that *M*. *hepatizon* and *M*. *disjuncta* were distinct from the rest, without the power to separate *M*. *agnata* and *M*. *stygia* due to their chemical similarity. Orthogonal partial least–squares to latent structures–discriminate analysis (OPLS-DA), however, successfully distinguished *M*. *agnata* and *M*. *stygia* by identifying statistically significant metabolites, which lead to class differentiation. This work has demonstrated the potential of DNA barcoding, chemical profiling and chemometrics in identification of *Melanelia* species.

## Introduction

The lichen-forming fungi (ascomycetes) in the genus *Melanelia* belong to the family Parmeliaceae, and these lichen taxa typically consist of brown and black foliose thallus, while the lobes are usually narrow, elongated and flat to convex or concave [1,2]. This genus was reported to contain *ca* 40 taxa with *M*. *stygia* as the type species [1]. However, according to numerous extensive systematic revisions, the number of species in this genus was reduced to four: *M*. *stygia*, *M*. *hepatizon*, *M*. *agnata* and *M*. *pseudoglabra* [3–13]. The rest of the formerly assigned *Melanelia* species are circumscribed into other genera using molecular data, including *Melanohalea* [3,10,12], *Melanelixia* [3,10], *Cetrariella* [4], *Montanelia* [8–10] and *Nephromopsis* [11]. A recent phylogenetic study [4] supports the placement of the genus *Melanelia* in the cetrarioid group, instead of the parmelioid group where the genus was originally placed.

Lichens in Parmeliaceae are prolific producers of bioactive compounds, which have also been widely used for chemotaxonomic purposes [14,15]. However, the secondary metabolite profiles of the genus *Melanelia* have not been fully studied apart from thin layer chromatography profiling [16,17]. Rapid instrumental development has rendered liquid chromatography coupled to mass spectrometry (LC-MS) a useful tool to investigate lichen secondary metabolites for ecological [18], biological [19] and chemotaxonomical purposes [20,21]. Moreover, chemometrics using principal component analysis (PCA) of large datasets has shown great potential in the classification of lichen taxa and discovery of novel secondary metabolites [22].

In total four species of the genus *Melanelia* have been reported to grow in Iceland: *M*. *agnata*, *M*. *disjuncta*, *M*. *hepatizon* and *M*. *stygia* [17]. Although *M*. *disjuncta* now belongs to the genus *Montanelia* [8], we decided to include that taxon in our study. Due to high morphological similarity and limited data on chemical characters, phenotypic identification of specimens of those taxa in Iceland can be very challenging. DNA barcoding using the nuclear ribosomal internal transcribed spacer region (nrITS) has demonstrated considerable discriminatory power for a wide range of fungal species [23,24], which could be applied to the *Melanelia* species. Therefore, the aim of the study was to identify specimens of the genus *Melanelia* in Iceland using DNA barcoding, to investigate the chemical diveristy of those lichen taxa and to test the utility of chemometrics for specimen discrimination.

## Materials and methods

### Taxon sampling

Previously reported *Melanelia* species in Iceland belong to two groups: the genus *Melanelia* in the cetrarioid group, and *Montanelia disjuncta* (Basionym: *Melanelia disjuncta*) in the parmelioid group. We have sampled 116 specimens representing species in the genera *Melanelia* and *Montanelia*, including 21 Icelandic specimens (S1 Table). The samples of the genus *Melanelia* include: *M*. *agnata* (7 specimens), *M*. *hepatizon* (22 specimens) and *M*. *stygia* (10 specimens). The species *M*. *pseudoglabra* is missing in our sampling, since it has not been found in Iceland. There are no relevant molecular data available concerning this species, except for a report of a new tridepside in the Australian *M*. *pseudoglabra* population [25]. Samples of the genus *Montanelia* include: *M*. *disjuncta* (33 specimens), *M*. *panniformis* (10 specimens), *M*. *sorediata* (7 specimens) and *M*. *tominii* (27 specimens). A number of *Montanelia* species are not present in Iceland, and we used nrITS sequence data of those *Montanelia* species outside Iceland covering multiple locations, to maximize and represent intraspecific and interspecific genetic variation. Reference sequences not covering the whole nrITS region (ITS1, 5.8S and ITS2) were discarded, and finally a matrix of 116 nrITS sequences was obtained from GenBank (95 sequences) and our samples (21 sequences). Voucher information and GenBank accession numbers for genetic sequences used for this study are provided in S1 Table.

### LC-MS metabolite profiling and multivariate statistical data analysis

Metabolite profiling was carried out for the discrimination of Icelandic specimens. Air-dried lichen thallus (ca. 30 mg) was weighed and ground into powder under liquid nitrogen. Lichen secondary metabolites were extracted three times by acetone maceration under shaking at ambient temperature. Extracts were combined and evaporated with nitrogen gas flow. Dried residues were then solubilized in HPLC-grade acetonitrile, diluted into 0.1 mg/mL and filtered before ultra-performance liquid chromatography-electrospray ionization-quadrupole time-of-flight mass spectrometry (UPLC-ESI-QTOF/MS) analysis. Residual thallus powder was air-dried in a fume hood under ventilation and stored at room temperature for DNA extraction.

The profiling of the lichen acetone extracts was performed using a Waters Acquity UPLC system, coupled to Synapt G1 QTOF/MS (Waters corp., Milford, MA, USA) equipped with ESI interface. The separation of lichen acids was performed on a Waters ACQUITY UPLC BEH C_18_ (2.1 mm x 100 mm 1.7 μm) column, which was maintained at 40°C in a column oven. The injection volume was 2 μL. Mobile phase consisted of solvent A: H_2_O with 0.1% formic acid and solvent B: CH_3_CN with 0.1% formic acid, at a flow rate of 0.45 mL/min. Linear gradient elution conditions were as follows: 15% B, for 0–4 min; 15% to 70% B, at 4–5.5 min; holding at 70% B, for 5.5–10.5 min; 70% to 100% B, at 10.5–12 min; 0 to 85% A, at 12–12.5 min; 85% A, for 12.5–13.5 min. Ionization was performed in the negative ESI mode. Parameters of the ESI source were: capillary voltage 3.5 kV; cone voltage 15 V; source temperature 120°C; desolvation temperature 450°C at a flow rate of 700 L/h (N_2_); cone gas flow rate at 50 L/h. Ions within the range of 50 to 1500 mass to charge ratio (m/z) were scanned by the mass analyzer. Pooled samples (i.e. combination of sample extracts) were analyzed across the whole run and used as quality control. MassLynx 4.1 software (Waters corp., Milford, MA, USA) was used for data acquisition. All samples were run in triplicates.

MS spectra were aligned and normalized using MakerLynx 4.1 (Waters corp., Milford, MA, USA). Collection parameters were set as 150 counts, mass window 0.05 Da and retention time window 0.2 min. Replicate percentage value was set at 50%. Normalized data were introduced into EZinfo software (Sartorius Stedim Data Analytics AB, Umeå, Sweden) for principal component analysis (PCA) and also orthogonal partial least-squares to latent structures—discriminate analysis (OPLS-DA). Compounds were identified by the evaluation of their MS/MS spectra and fragmentation patterns as well as comparison with isolated pure compounds, earlier published data, or searching databases (Metlin and ChemSpider).

### Molecular analysis

#### DNA extraction, PCR and sequencing

Lichen genomic DNA was isolated from dried lichen powder after acetone extraction using the CTAB method [26]. DNA extracts were stored in 30 μL 1×TE buffer (pH 8.0) and quantified using Quant-iT Picogreen^™^ assay according to the manufacturer’s instructions. The nrITS region was amplified by polymerase chain reactions (PCRs) using primer pair ITS1F [27] and ITS4 [28]. Each amplification reaction (25 μL) contained 1×standard Taq reaction buffer for nrITS, 200 μM dNTPs, 0.2 μM forward and reverse primer, 1.25 units of Taq DNA polymerase (New England Biolabs), 1 or 3 μL DNA template, and PCR-grade water. PCR cycling conditions were as follows: initial denaturation at 94°C for 3 min, 31 cycles of 94°C for 40s, 54°C for 40s and 68°C for 1min, final extension at 68°C for 7min and cooling down to 4°C. PCR amplicons were purified using EXO-SAP (Fermentas) following manufacturer’s instruction. Purified PCR products were sent for Sanger sequencing by Macrogen Inc., using the same sets of primers for PCRs.

#### Multiple sequence alignment

Sequences were primarily aligned using PhyDE-Phylogenetic Data Editor 0.9971 and MUSCLE [29], followed by manual refinement. Introns in the nr ITS matrix were removed together with ambiguous regions at both ends. Sequences not covering the whole nrITS region were discarded. Gaps were treated as missing characters. For the calculation of intraspecific p-distances, sequences are re-aligned using the same approach for each species.

#### DNA barcoding analysis

As suggested for barcoding analysis [30], the construction of distance matrices in this study employed uncorrected p-distance with pairwise deletion of gaps. Genetic distances were calculated using MEGA 6.0 [31]. Distance data were displayed with a dot-plot for each species, in which the nearest interspecfic distance is plotted to the furthest intraspecific distance. A barcoding gap is present when the smallest interspecific distance is larger than the maximum intraspecific distance. Distinct species are recognized by the presence of barcoding gaps. A summary of p-distance data was made using the oneline version of Automatic Barcoding Gap Discovery (ABGD) [32] for each genus.

In order to graphically summarize genetic distance data and to assess if sequence data form species-specific clades, a phenetic neighbor joining (NJ) clustering analysis was performed using MEGA 6.0 [31]. The construction of NJ tree employed a p-distance model with pairwise deletion of gaps. Nodal support values were calculated using 1000 bootstrap replicates.

#### Phylogenetic analysis

A fungal nrITS gene tree was constructed using both Bayesian inference and Maximum likelihood methods. In Bayesian inference analysis, the molecular evolution model (GTR+I+G) for the nrITS locus was estimated by MrModeltest v2.3 [33] according to Akaike Information Criteria. Bayesian Inference of the phylogenetic tree using Markov Chain Monte Carlo sampling [34] was performed with MrBayes v3.2.6 [35]. Phylogenetic analysis was run on four chains for 10 million generations, with the first 25% trees discarded. With the same alignment, a maximum likelihood analysis was carried out on RAxML GUI 1.3 [36] using the GTRGAMMA model. Support values were estimated by 1000 bootstrap pseudoreplicates. *Protoparmelia ochrococca* was selected as the outgroup, since it is a sister species to Parmeliaceae [5,37,38]. Phylogenetic trees were visualized using FigTree 1.4.2. Posterior probability over 0.95 and bootstrap support value over 70 of each clade were regarded as significant.

## Results and discussion

### LC-MS chemical profiling of *Melanelia* lichens and identification of major lichen acids by mass spectrometry

**Fig 1:**
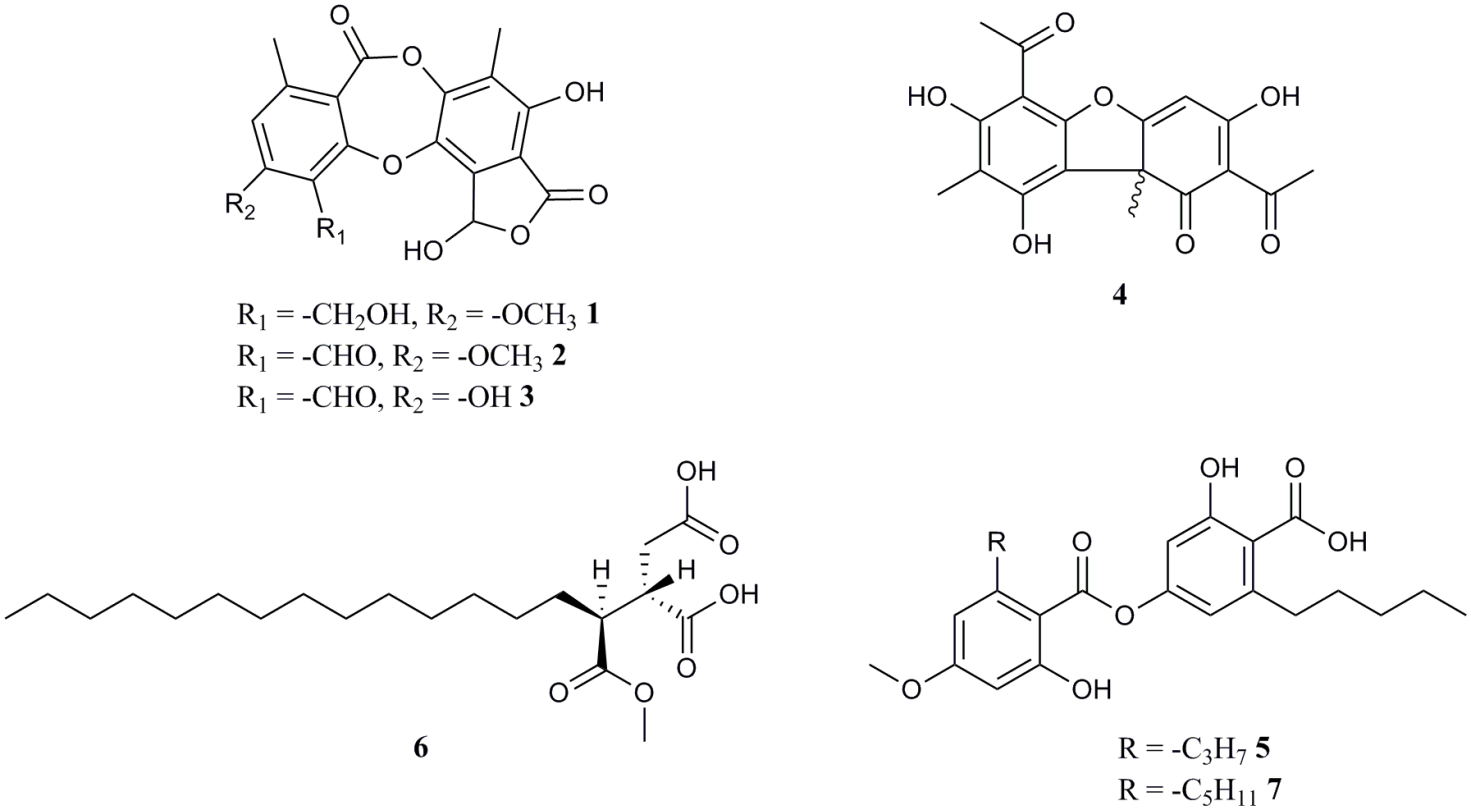
Chemical structures of major compounds in Icelandic *Melanelia* lichens. Compounds include cryptostictic acid **1**, stictic acid **2**, norstictic acid **3**, usnic acid **4**, stenosporic acid **5**, rangiformic acid **6** and perlatolic acid **7**.

**Fig 2:**
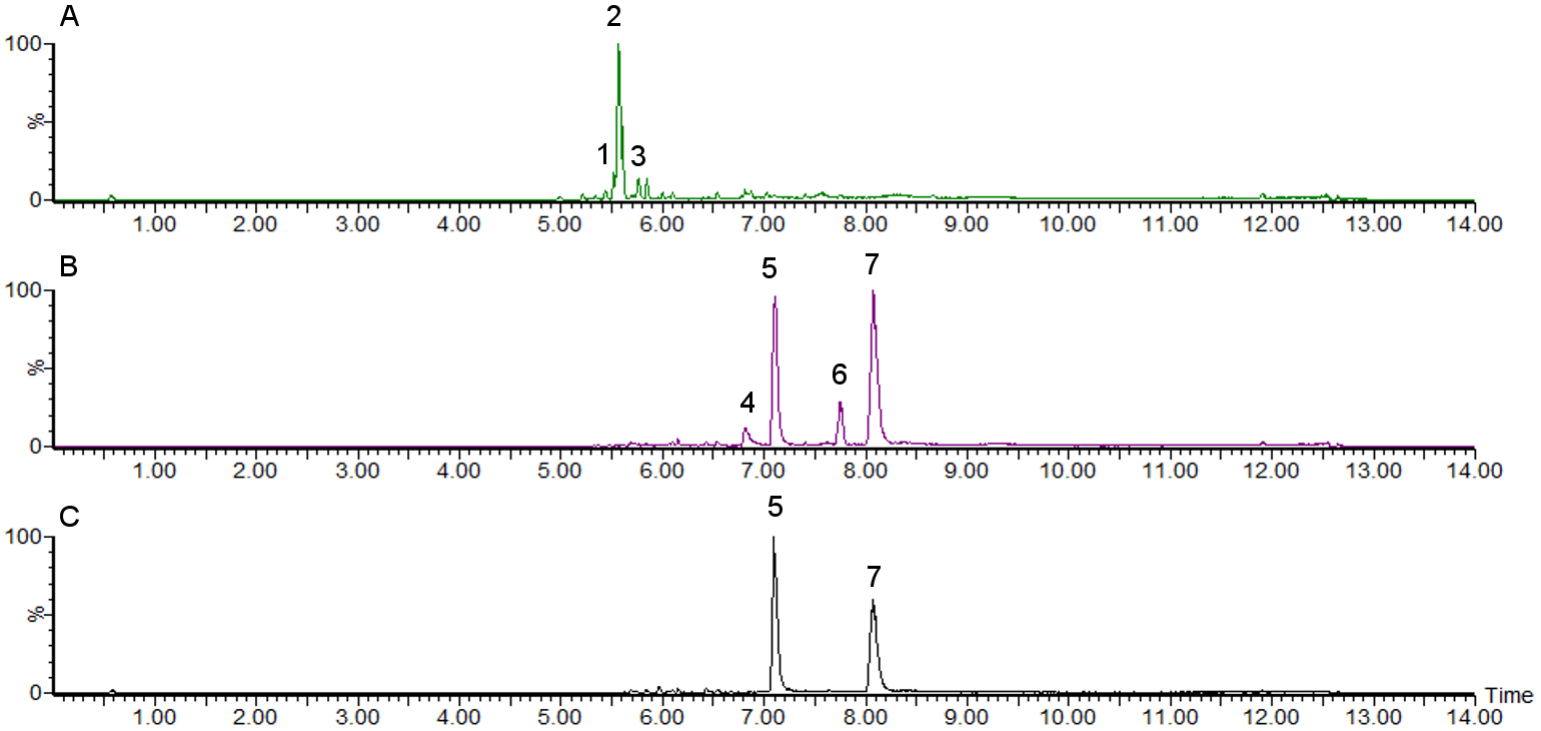
Base peak MS chromatograms of lichen acetone extracts in negative ion mode. (A) MS chromatogram of *Melanelia hepatizon* containing cryptostictic acid **1**, stictic acid **2** and norstictic acid **3**. (B) MS chromatogram of one *Montanelia disjuncta* chemotype that contains usnic acid **4**, stenosporic acid **5**, rangiformic acid **6** and perlatolic acid **7**. (C) MS chromatogram of the other *Montanelia disjuncta* chemotype without usnic acid **4** and rangiformic acid **6**. Chromatograms of *Melanelia stygia* and *M*. *agnata* are not shown since no major lichen acids were detected.

LC-MS chemical profiling analysis has not been reported on *Melanelia* species before, and the only chemical analysis available is thin layer chromatography (TLC) profiling indicating the presence of some depsidones in *M*. *hepatizon* and certain chemotypes of *M*. *stygia* [17]. Detected major lichen compounds in this study were listed in Fig 1 as compounds **1**–**7**. Fig 2 shows the base peak mass chromatograms of acetone extracts of *Melanelia* lichens.

Interestingly, Icelandic *M*. *agnata* and *M*. *stygia* specimens do not contain any major lichen acids analyzed in negative ion mode (chromatograms not shown), although it has been reported that *M*. *stygia* collected from China contains depsidones, such as fumarprotocetraric acid and protocetraric acid [39]. On the other hand absence of lichen acids in European *M*. *stygia* taxa has also been reported [17]. TLC analysis of Icelandic *M*. *agnata* organic extracts has shown the absence of major lichen acids [16] which is in agreement with our findings. Two chemotypes of Icelandic *Montanelia disjuncta* taxa have been discovered: one contains usnic acid and rangiformic acid, while the other one does not. Notably, the discovery of usnic acid **4** in the chemotype of *M*. *disjuncta* disagreed with a former study [40], which indicates the abscence of usnic acid in *M*. *disjuncta* related lichen taxa. The detection of usnic acid in our study is not expected to be from outside contamination, because no usnic acid-containing lichen samples were running in the same batch. Additionally, it is apparent from the chromatograms that *M*. *hepatizon* has the depsidone stictic acid **2** as the predominant metabolite, while *M*. *disjuncta* has depsides stenosporic acid **5** and perlatolic acid **7** as major metabolites, which corresponds to the TLC profiling results [17].

Metabolites have been characterized in previous studies and were therefore identified by their molecular mass, fragmentation patterns and comparison with reference MS data [18,20,41]. A list of major metabolites is presented in Table 1.

**Table 1:** Major lichen metabolites (1–7) and unknown minor compounds (a-c) in Icelandic *Melanelia* taxa.

No.	T_R_ (min)	[M-H]^-^(m/z)	Molecular formula	Characteristic product ion (m/z)	Compound	Lichen
**a**	5.43	-	-	357.0674, 313.0791, 269.0962	unknown	*M*. *hepatizon*
**1**	5.52	387.0721	C_19_H_16_O_9_	343.0841, 299.0995, 284.0786	Cryptostictic acid	*M*. *hepatizon*
**2**	5.57	385.0523	C_19_H_14_O_9_	341.0630, 297.0795, 267.0325	Stictic acid	*M*. *hepatizon*
**b**	5.60	-	-	373.0614, 329.0663	unknown	*M*. *hepatizon*
**3**	5.76	371.0421	C_18_H_12_O_9_	327.0511, 283.0695, 243.0386, 227.0433	Norstictic acid	*M*. *hepatizon*
**c**	5.84	425.0843	C_22_H_18_O_9_	381.0982, 337.1136, 322.0933	unknown	*M*. *hepatizon*
**4**	6.82	343.0832	C_18_H_16_O_7_	328.0633, 259.0599, 231.0655	Usnic acid	*M*. *disjuncta*
**5**	7.11	415.1794	C_23_H_26_O_7_	223.1034, 205.0993, 179.1152	Stenosporic acid	*M*. *disjuncta*
**6**	7.75	385.2618	C_21_H_38_O_6_	353.2296, 309.2544, 265.2555	Rangiformic acid	*M*. *disjuncta*
**7**	8.08	443.2073	C_25_H_32_O_7_	223.1049, 205.0932, 179.1187	Perlatolic acid	*M*. *disjuncta*

Compounds were detected by ESI-MS in negative ion mode. Their chromatographic data and characteristic MS fragmentation ions are provided. Molecular formulas were calculated by elemental analysis function by MassLynx 4.1.

**Fig 3:**
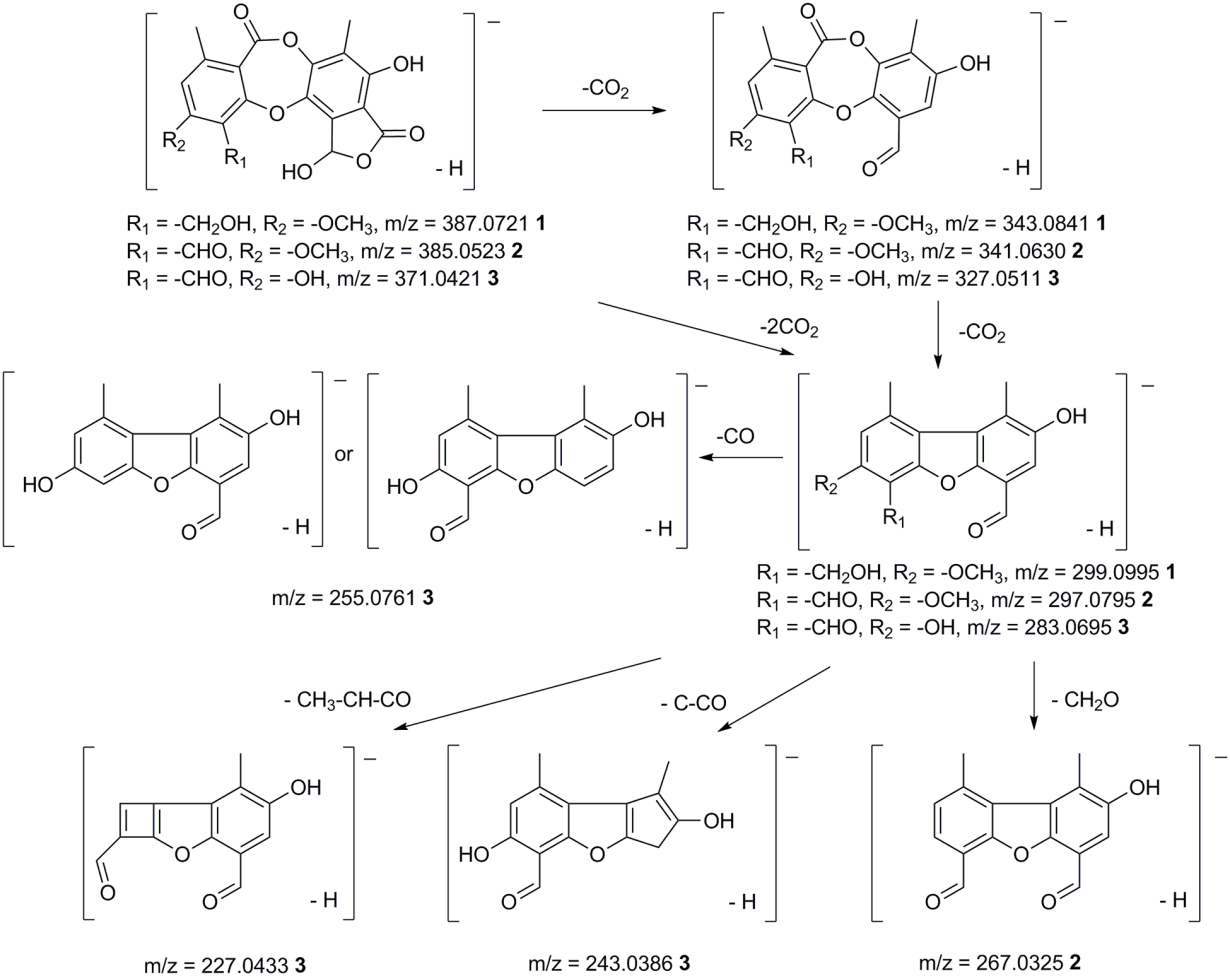
MS fragmentation patterns of depsidones in the lichen *Melanelia hepatizon*. Compounds include cryptostictic acid **1**, stictic acid **2** and norstictic acid **3**.

**Fig 4:**
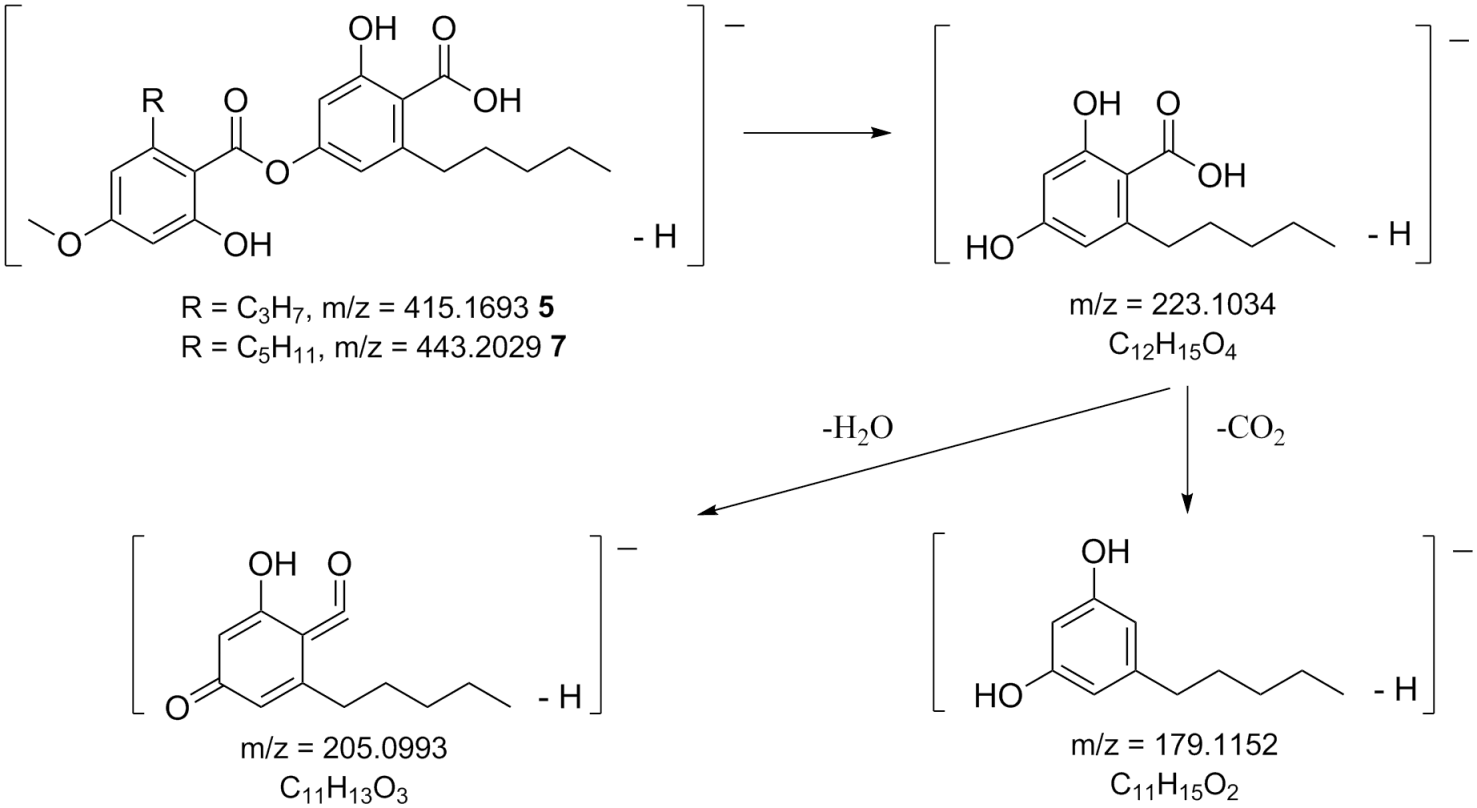
MS fragmentation patterns of depsides in the lichen *Montanelia disjuncta*. Compounds are stenosporic acid **5** and perlatolic acid **7**. Both shared the same fragment ions.

**Fig 5:**
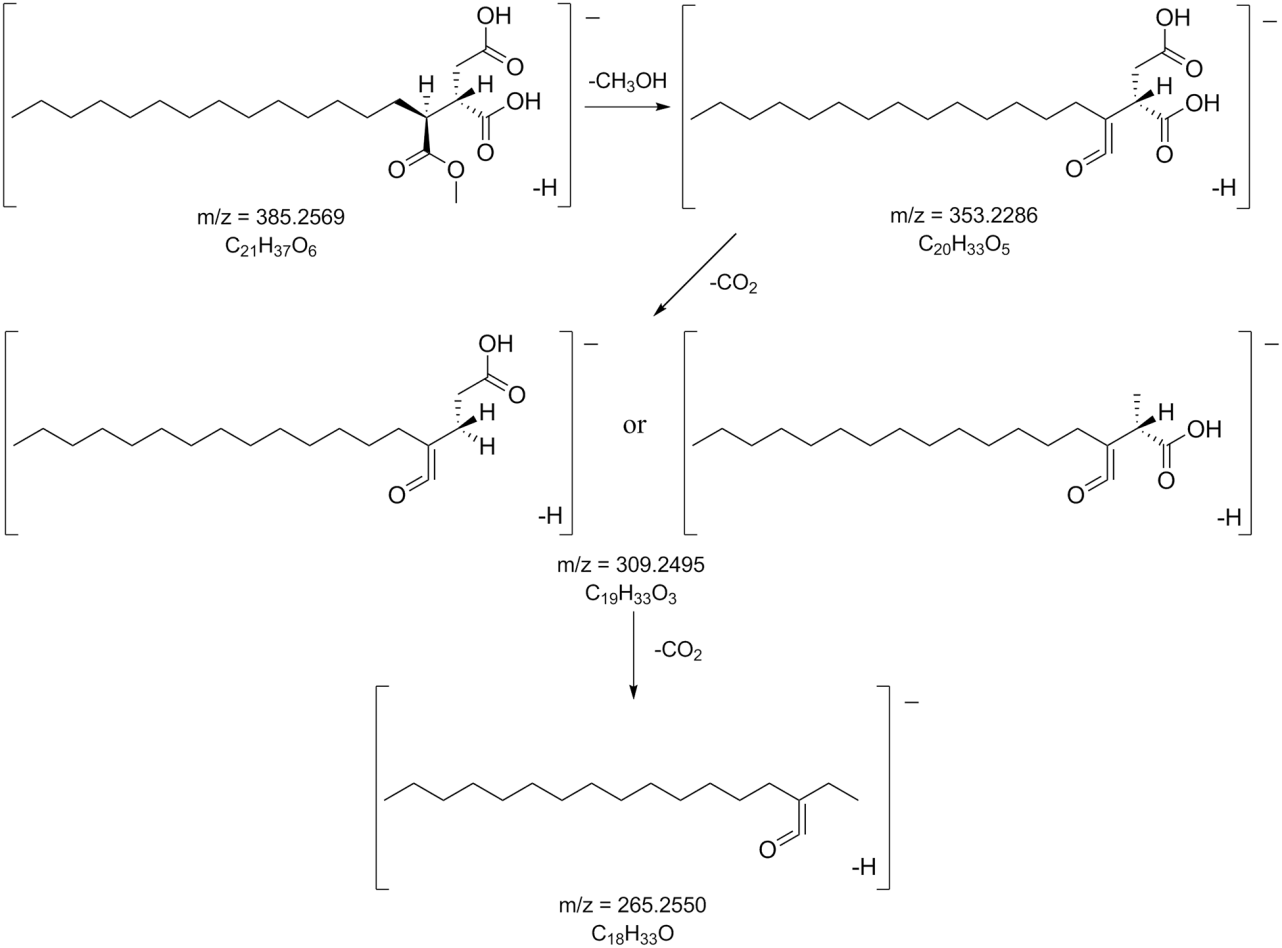
MS fragmentation pattern of rangioformic acid. Rangiformic acid **6** was found in one chemotype of Icelandic *Montanelia disjuncta* taxon, as shown in Fig 2B.

The consecutive loss of CO_2_ in the lactone rings is characteristic for depsidones in *Melanelia hepatizon*, as deduced from their MS^2^ spectra (S1 Fig). The major fragmentation pathway (Fig 3) agrees with those proposed by Parrot et al. [20]. MS fragmentation of fatty depsides in *Montanelia disjuncta* showed typical cleavage of an ester bond between two aromatic rings, followed by further cleavage of substitutes in the aromatic moiety (Fig 4). Rangiformic acid **6** was identified by its deprotonated molecular ion at m/z = 385 and fragmentation pathway loosing a CH_3_OH and two CO_2_ (Fig 5). MS^2^ spectra of detected depsides, usnic acid and rangiformic acid are shown in S2, S3 and S4 Figs respectively.

**Fig 6:**
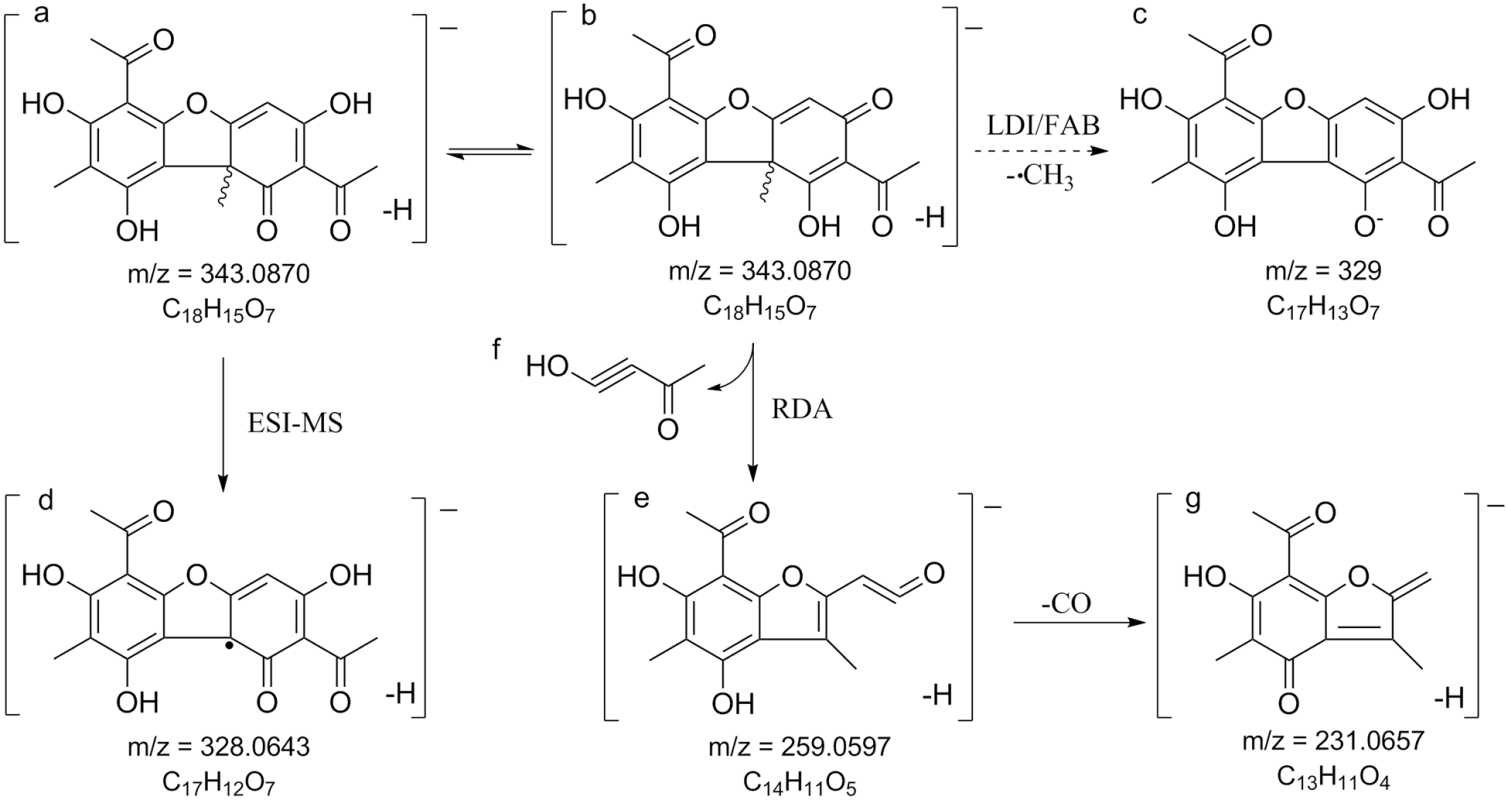
MS fragmentation pathway of usnic acid. Usnic acid was detected in one chemotype of Icelandic *Montanelia disjuncta* taxon. Structure a, b, d, e and f are characteristic fragment ions in MS^2^ spectrum (S3 Fig). Structure b is a resonance contributor of structure a but not a true structure of usnic acid. Structure c is only reported in LDI or FAB-MS. (Abbreviation: RDA: retro-Diels—Alder reaction; LDI/FAB: laser desorption ionization/fast atom bombardment).

Even though usnic acid **4** is one of the most studied lichen compounds, its negative ion mode mass fragmentation pathway has rarely been reported [42]. Here we have established the complete MS and MS^2^ usnic acid mass spectra in S3 Fig as well as the deduced fragmentation pathway in Fig 6, which has also been verified by authentic standard. The ESI-MS spectrum of usnic acid usually shows an adduct ion of m/z = 709, which corresponded to [2M – 2H + Na^+^]^-^. Fragmentation of usnic acid in negative ion mode seems to depend on the MS interface. Ionization in either fast atom bombardment [43] or laser desorption ionization [19] interface gives rise to the major product ion at m/z = 329 [M—CH_3_], which is found to be the only product ion present in negative ion mode [41]. However, this study and the one by [42] using ESI found the major product ion at m/z 328 [M—CH_4_]^-^ instead. Further fragmentation of usnic acid molecular ion involved the retro-Diels-Alder reaction [42][41], where the resonance contributor (structure b, Fig 6) of usnic acid was cleaved to a diene (structure e, Fig 6) and a dienophile (structure f, Fig 6).

### Multivariate analysis of LC-MS chemical profiles for specimen discrimination

**Fig 7:**
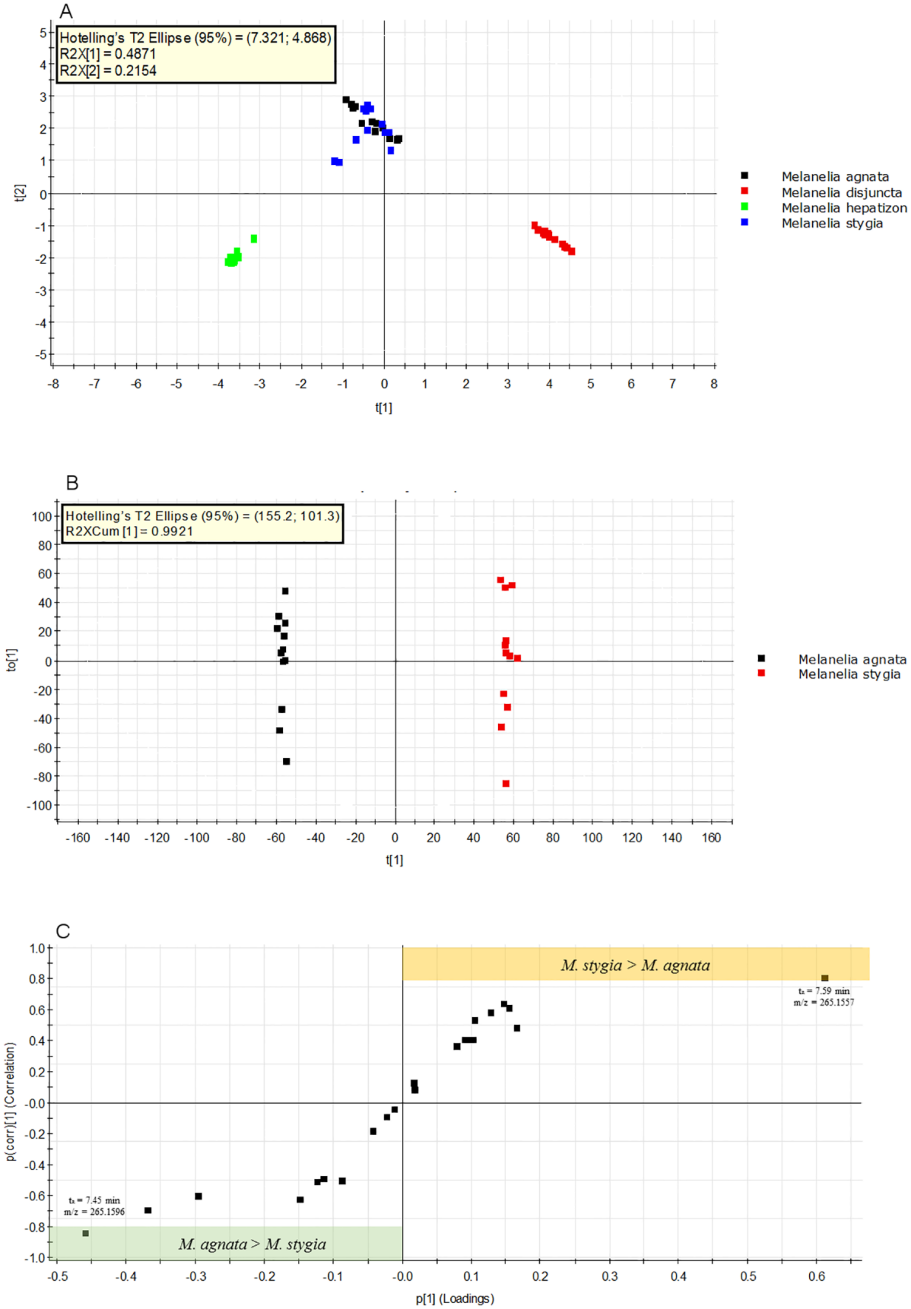
Multivariate analysis of LC-MS metabolite data. (A) PCA plot of chemical profiles of Icelandic *Melanelia* lichens, where *M*. *agnata* and *M*. *stygia* are clustered. (B) OPLS-DA plot shows the separation of the two *Melanelia* taxa. The metabolome of *M*. *agnata* and *M*. *stygia* can be differentiated with a high level of prediction value: R^2^Y(cum) = 1, Q^2^(com) = 0.99. (C) Loading S-plot from LC-MS data of *M*. *agnata* and *M*. *stygia*. Cut-off values of p(corr) < |0.8| were selected to designate the metabolites contribuiting significantly to the overall difference (area in color) between *M*. *agnata* and *M*. *stygia*. Two metabolites were thus identified from each species.

Using LC-MS chemical profiling, a complex metabolite dataset has been obtained, which requires chemometrics tools to summarize, visualize and interpret. As an unsupervised multivariate analysis method, PCA gives an overview of the difference of metabolite profiles. As shown in the PCA plot in Fig 7, three independent groups were recognized: *Melanelia hepatizon*, *Montanelia disjuncta* (*Melanelia disjuncta*), and a cluster of *Melanelia stygia* and *Melanelia agnata*.

Principal component 1 and component 2 accounted for 48.7% and 21.5% of total variance of normalized LC-MS data, respectively. It is conceivable that *M*. *hepatizon* and *Montanelia disjuncta* contained species-specific metabolite biomarkers as well as disctinct chromatograms, as shown in Table 1. However, *M*. *agnata* and *M*. *stygia* contained no apparent markers. Although PCA provides a holistic overview, it could not distinguish *M*. *agnata* from *M*. *stygia*. It is known that PCA tends to summarize the trends of data, but may deviate from addressing the question of interest due to experimental variations [44].

In this study we adopted OPLS-DA, as a supervised method, to see if this approach could identify potential markers or chemotypic differences between the two species, which contained metabolites in very low concentrations. As shown in Fig 7B, clear group separation was achieved in the OPLS-DA model, which was supported by the predictive ability Q^2^(Y) = 100% and total explained variance R^2^(X) = 99%. Applying the cut-off values for the predictive ability as Q2 < |0.8|, we identified two minor metabolits that contributed most to the separation of *M*. *agnata* and *M*. *stygia*: 7.59 min, m/z = 265.1557 in *M*. *stygia* and 7.45 min, m/z = 265.1596 in *M*. *agnata*. The combination of OPLS-DA with S-plot in this study could handle complex metabolite data and was able to propose the metabolites of discriminatory power for sampled Icelandic taxa. This approach has previously been used in phenotypic differentiation of aspens, tea cultivars, insects and bacterial strains by extracting metabolites of statistical and potentially biological interest [44–47].

It should be emphasized that the discriminatory power of the two compounds identified from S-plot (Fig 7C) can not be considered to have chemotaxonomic value until their presence has been confirmed from worldwide sampling. Lichens can be inconsistent in the production of lichen compounds, reflected by intraspecific chemotypes spanning major lichen-forming fungal lineages [48–50]. Quantitative and qualitative chemical variations can be caused by a number of environmental factors, such as sunlight [51] and habitat ecology [52]. Therefore, a distinct chemical profile of a certain lichen taxon can not be independently used for lichen taxonomy, although it can be useful in correlation with other characteristics, such as morphology and molecular data [53]. This study has demonstrated the use of correlated chemical and molecular data for specimen identification and discrimination for Icelandic lichen taxa.

The chemical analysis in this study was carried out on dry lichen herbarium materials collected from 1997 to 2014. The results show clustering of specimens belonging to certain lichen species irrespective of age, indicating reproducibility and stability of the lichen compounds under dry storage conditions. Therefore, the use of dry lichen material does not seem to interfere with the study of chemical relatedness of *Melania* species, although we still recommend to include fresh material for comparison in future studies.

### DNA barcoding for specimen identification of reported *Melanelia* species in Iceland

The nrITS alignment file contained 116 sequences with 495 characters (161 variable sites), with 21 newly generated sequences. A summary of intraspecific p-distances, alignment length, number of specimens and haplotypes for reported *Melanelia* species in Iceland is presented in Table 2.

**Table 2:** Genetic distances, alignment length and number of specimens (haplotypes) for the genera *Melanelia* and *Montanelia*.

Species	Number of sequences	Alignment length (bp)	Mean±SD	Range
***Melanelia agnata***	7 (4)	493	0.0136±0.0035	0.0–0.0244
***Melanelia stygia***	10 (3)	493	0.0195±0.0044	0.0–0.0346
***Melanelia hepatizon***	22 (9)	491	0.0131±0.0026	0.0–0.0307
***Montanelia disjuncta***	33 (15)	494	0.0103±0.0023	0.0–0.0306
*Montanelia tominii*	27 (11)	494	0.0170±0.0030	0.0–0.0407
*Montanelia panniformis*	10 (4)	492	0.0108±0.0024	0.0–0.0407
*Montanelia sorediata*	7 (2)	490	0.0049±0.0022	0.0–0.0102

Values following means are standard deviations. Previously reported *Melanelia* species in Iceland are in bold.

**Fig 8:**
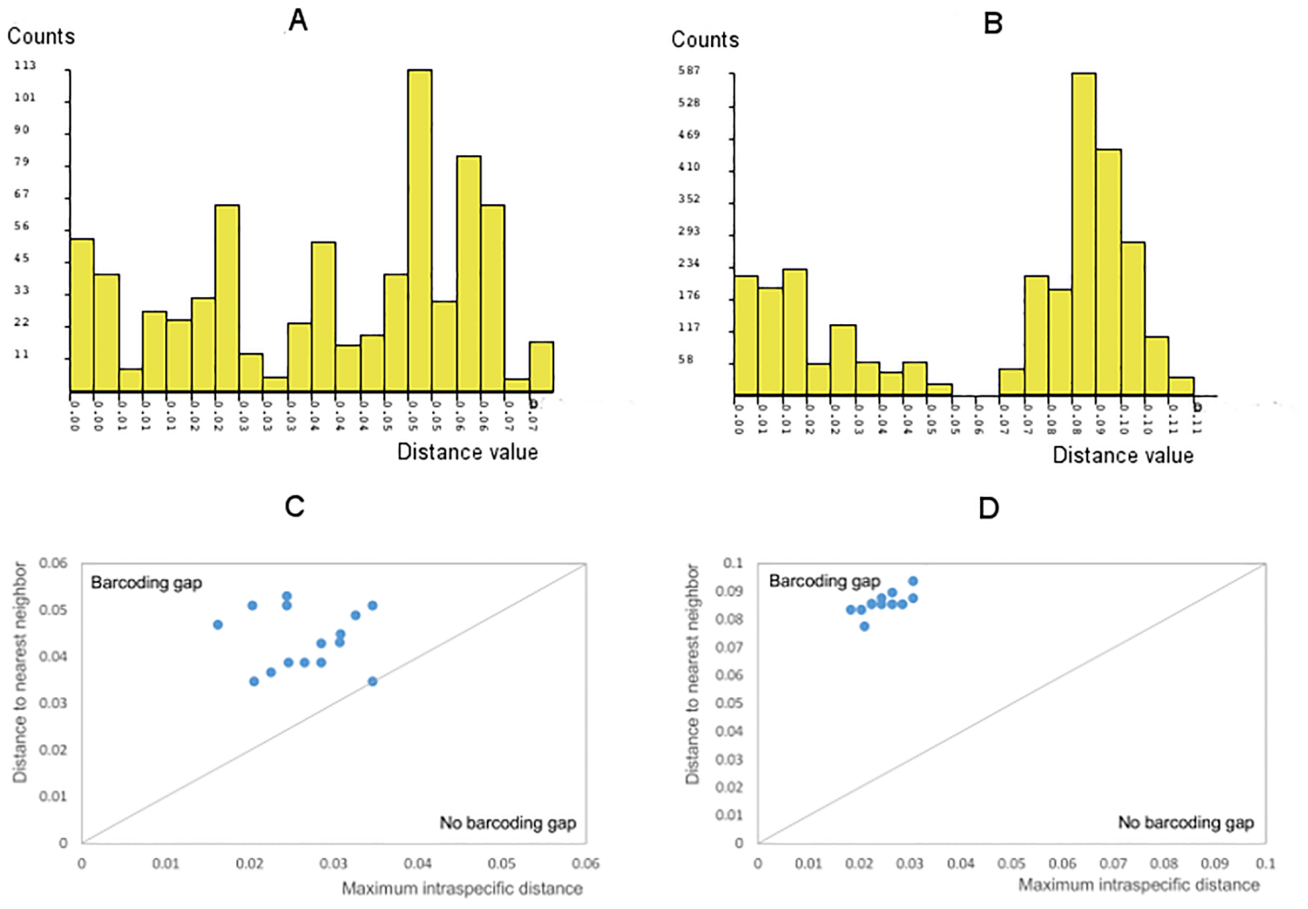
Genetic distance histograms and barcoding gap analysis for reported *Melanelia* species in Iceland. (A and B) p-Distance histogram for the genus *Melanelia* and *Montanelia*, respectively; (C and D) Barcoding gap analysis for *Melanelia* species and *Montanelia disjuncta*.

**Fig 9:**
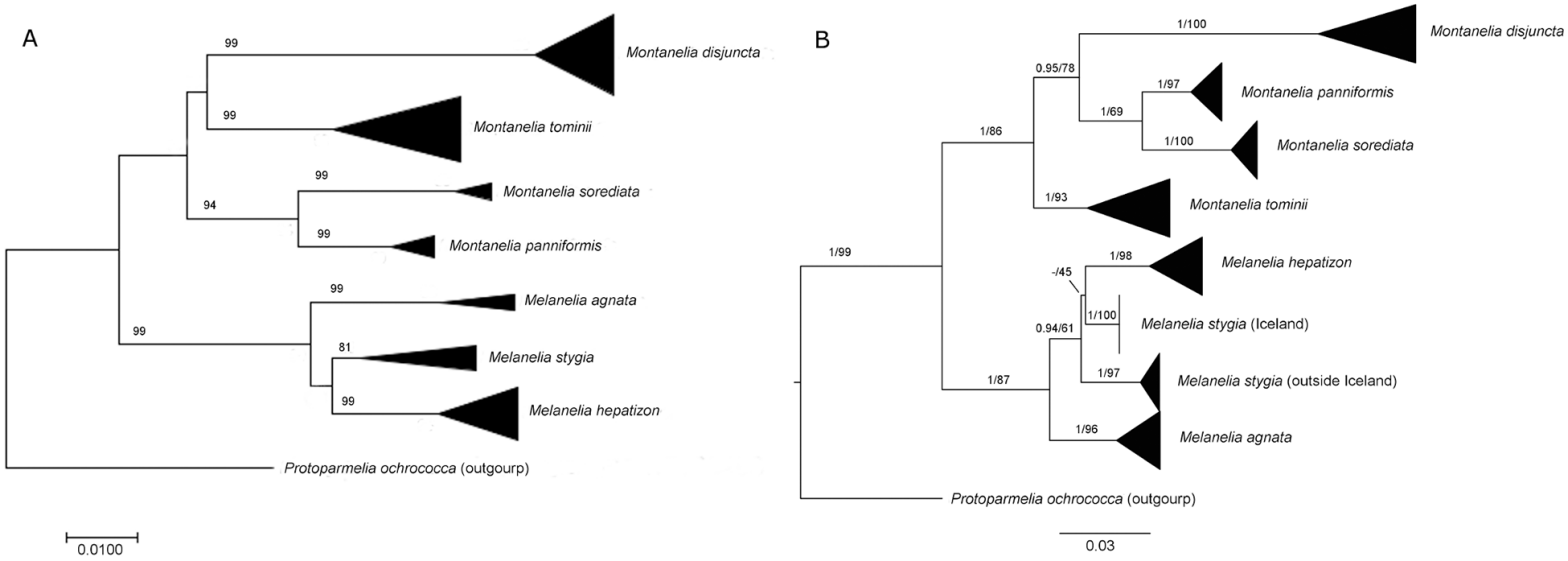
Fungal nrITS gene tree obtained from 116 *Melanelia* and *Montanelia* specimens. (A) Neighbor-joining tree, bootstrap values over 80% are labelled on the branches; (B) Maximum-likelihood tree, posterior probability/bootstrap values are labelled on branches.

Among the reported species in Iceland, the highest mean p-distances were found in *M*. *stygia*, while the lowest in *Montanelia disjuncta*. Distance histograms for each genus and barcoding gap analysis results are presented in Fig 8. For the genus *Melanelia*, even though an overlapping between intra- and interspecific distances was found (Fig 8A), barcoding gaps were present for all *Melanelia* species (Fig 8C). Notably, for Icelandic *M*. *stygia* specimens, the minimum interspecific genetic distance (0.0347) is only slightly higher than the maximum intraspecific distance (0.0346). Together with its high mean genetic distance among sampled *M*. *stygia* specimens, our results support the potential for previously unrecognized lineages in the species *M*. *stygia*. The suggestion for potentially new species lineages in the genus *Melanelia* has been raised earlier by another DNA barcoding study [54], where high intraspecific genetic distances were found. The genus *Montanelia* showed a general distance gap for all species at the p-distance range 0.05–0.07 (Fig 8B), and a barcoding gap is also present for the species *Montanelia disjuncta* (Fig 8D). All previously reported *Melanelia* species in Iceland formed significant (bootstrap value > 80%) monophyletic clades in the p-distance-based NJ tree (Fig 9A). NJ tree with all specimens is provided in S5 Fig. However, the monophyletic clade of the species *M*. *stygia* was not recovered in the ITS gene tree (Fig 9B) constructed with both Baysian Inference (S6 Fig) and Maximum Likelihood methods (S7 Fig).

## Conclusions

This study used chemical profiling and DNA barcoding to identify and discriminate specimens of the reported *Melanelia* lichens in Iceland. Major lichen acids were identified and the metabolite profiles provided a chemotaxonomic basis for the formerly confusing lichen-forming fungal genus *Melanelia*. The segregation of the genus *Montanelia* from *Melanelia* is supported by our metabolite profiling data as well as phylogenetic results. Chemotypes of Icelandic lichen taxon *Montanelia disjuncta* were discovered by LC-MS profiling, and reported for the first time. Icelandic *Melanelia stygia* and *M*. *agnata* contained no major lichen acids and were separated by minor or trace metabolites using sensitive MS detection and multivariate data analysis, underlining the power of MS and chemometric analysis in specimen discrimination.

## Supporting information

S1 Table Voucher information and sequence accession numbers. (PDF)

S1 Fig MS^2^ spectra of depsidones in the lichen *Melanelia hepatizon*. (A) cryptostictic acid **1**. (B) stictic acid **2**. (C) norstictic acid **3**.
(PDF)

S2 Fig MS^2^ spectra of depsides in the lichen *Montanelia disjuncta*. (A) stenosporic acid **5**. (B) perlatolic acid **7**.
(PDF)

S3 Fig MS and MS^2^ spectra of usnic acid in one chemotype of the lichen *Montanelia disjuncta*. (A) MS spectrum of usnic acid **4** showing the adduct ion at m/z 709.1456 and molecular ion at m/z 343.0847. (B) MS^2^ spectrum of the usnic acid molecular ion.
(PDF)

S4 Fig MS^2^ spectrum of rangiformic acid. (PDF)

S5 Fig Neighbor-joining nrITS gene tree from 116 specimens representing all *Melanelia* and *Montanelia* species. Bootstrap values (%) over 50 are labelled above branches.
(PDF)

S6 Fig Baysian nrITS gene tree from 116 specimens representing all *Melanelia* and *Montanelia* species. Posterior probabilities over 0.94 are labelled above branches.
(PDF)

S7 Fig Maximum likelihood nrITS gene tree from 116 specimens representing all *Melanelia* and *Montanelia* species. Bootstrap values (%) over 70 are labelled above branches.
(PDF)
