# Supplementary material for: DNA barcoding and LC-MS metabolite profiling of the lichen-forming genus Melanelia: Specimen identification and discrimination focusing on Icelandic taxa
Source: PLoS One. 2017 May 24;12(5):e0178012. doi: 10.1371/journal.pone.0178012 (PMC5443556; doi:10.1371/journal.pone.0178012)
Supplement: S1 Table — (PDF) [file pone.0178012.s001.pdf]

S1 Table. Voucher information and sequence accession numbers.

Genus	Species/lineage	Voucher Number	Country	State/Province/Zone	ITS GenBank Acession #
Melanelia	*hepatizon*	ESH-09B.164 (C)	Greenland	NWn, Siorapuluk	KF257943
Melanelia	*hepatizon*	ESH-09B.026 (C)	Greenland	NWn, Qaanaaq	KF257944
Melanelia	*hepatizon*	ESH-10B.014 (C)	Greenland	SEm, Tasilaq	KF257945
Melanelia	*hepatizon*	ESH-10A.019 (C)	Greenland	SWm, Nuuq	KF257946
Melanelia	*hepatizon*	ESH-08.036 (C)	Greenland	S, Qaqortoq	KF257947
Melanelia	*hepatizon*	ESH-08.170 (C)	Greenland	S, Igaliku	KF257948
Melanelia	*hepatizon*	ESH-08.263 (C)	Greenland	S, Narssarsuaq	KF257949
Melanelia	*hepatizon*	ESH-08.215 (C)	Greenland	S, Igaliku	KF257950
Melanelia	*hepatizon*	ESH-09.386 (C)	Greenland	SWm, Midgard	KF257951
Melanelia	*hepatizon*	ESH-09.324 (C)	Greenland	SWm, Frederikshab Isblink	KF257952
Melanelia	*hepatizon*	ESH-08.477 (C)	Greenland	S, Igaliku	KF257953
Melanelia	*hepatizon*	BC-9677 (LD)	Canada	British Columbia	AF141369
Melanelia	*hepatizon*	DNA-AT934 (LD)	Italy	Bozen Co.	AF451776
Melanelia	*hepatizon*	Wedin 6812 (UPS)	Sweden	unknown	DQ980016
Melanelia	*hepatizon*	LA30501	**Iceland**	Iau	**KY508674**
Melanelia	*hepatizon*	LA30676	**Iceland**	Ive	**KY508675**
Melanelia	*hepatizon*	LA30674	**Iceland**	Ive	**KY508676**
Melanelia	*hepatizon*	LA30675	**Iceland**	Ive	**KY508677**
Melanelia	*hepatizon*	LA30673	**Iceland**	Ive	**KY508678**
Melanelia	*hepatizon*	LA20781	**Iceland**	Ino	**KY508679**
Melanelia	*hepatizon*	LA31860	**Iceland**	Inv	**KY508680**
Melanelia	*hepatizon*	LA31861	**Iceland**	Inv	**KY963376**
Melanelia	*agnata*	ESH-09.478 (C)	Greenland	SW m, Jensens Nunatakker	KF257940
Melanelia	*agnata*	ESH-09.435 (C)	Greenland	SW m, Jensens Nunatakker	KF257941
Melanelia	*agnata*	ESH-07.464 (C)	Greenland	N, Constable Bugt	KF257942
Melanelia	*agnata*	LA29683	**Iceland**	Imi	**KY508672**
Melanelia	*agnata*	LA27562	**Iceland**	Ino	**KY963373**
Melanelia	*agnata*	LA30974	**Iceland**	Isu	**KY508673**
Melanelia	*agnata*	LA31859	**Iceland**	Ino	**KY963374**
Melanelia	*stygia*	ESH-08.036	Greenland	S, Qaqortoq	KF257954
Melanelia	*stygia*	ESH-08.478	Greenland	S, Igaliku	KF257955
Melanelia	*stygia*	FIN-9714 (LD)	Finland	Nyland	AF115763
Melanelia	*stygia*	DNA-AT922 (LD)	Italy	unknown	AF451775
Melanelia	*stygia*	Haikonen 20365	Finland	Enonkoski	AY611097
Melanelia	*stygia*	Hafellner 51658	Austria	Steiermark	AY611121
Melanelia	*stygia*	LA19972	**Iceland**	Iau	**KY508681**
Melanelia	*stygia*	LA28243	**Iceland**	Iau	**KY963375**
Melanelia	*stygia*	LA20775	**Iceland**	Iau	**KY508682**
Melanelia	*stygia*	LA16894	**Iceland**	Iau	**KY508683**
Montanelia	*disjuncta*	Spribille s.n. (GZU)	Canada	Yukon	KF257956
Montanelia	*disjuncta*	ESH-09B.363 (C)	Greenland	NWn, Siorapuluk	KF257957
Montanelia	*disjuncta*	ESH-08.304 (C)	Greenland	S, Igaliku	KF257958
Montanelia	*disjuncta*	ESH-09B.051 (C)	Greenland	NWn, Qaanaaq	KF257959
Montanelia	*disjuncta*	Harris 55589 (NY)	USA	Maine	KF257960
Montanelia	*disjuncta*	Bjerke WP286-2 (TLE)	Norway	Tromso	KF257961
Montanelia	*disjuncta*	Esslinger BP94-3 (TLE)	Canada	Yukon	KF257967
Montanelia	*disjuncta*	Esslinger 19403 (TLE)	USA	Alaska	KF257968
Montanelia	*disjuncta*	ESH-08.216 (C)	Greenland	S, Igaliku	KF257970
Montanelia	*disjuncta*	ESH-09B.323 (C)	Greenland	NWn, Siorapuluk	KF257971
Montanelia	*disjuncta*	Mayrhofer 13743	Austria	Steiermark	AY611077
Montanelia	*disjuncta*	Wedin 7143 (UPS)	Sweden	unknown	DQ980015
Montanelia	*disjuncta*	15512 (MAF)	India	Uttaranchal	GU994556
Montanelia	*disjuncta*	17227 (MAF-Lich)	UK	Scotland	JX974654
Montanelia	*disjuncta*	15512 (MAF-Lich)	India	Uttar Pradesh	KF257972
Montanelia	*disjuncta*	ESH-098.363	Greenland	Northwest	KP771825
Montanelia	*disjuncta*	ESH-08.304	Greenland	South	KP771826
Montanelia	*disjuncta*	ESH-09B.051	Greenland	Northwest	KP771827
Montanelia	*disjuncta*	ESH-08.216	Greenland	South	KP771832
Montanelia	*disjuncta*	ESH-09B.323	Greenland	Northwest	KP771833
Montanelia	*disjuncta*	Esslinger 19403	USA	Alaska	KP771831
Montanelia	*disjuncta*	Harris 52938	USA	Maine	KP771828
Montanelia	*disjuncta*	Lumbsch 2010/M7	USA	unknown	JX126181
Montanelia	*disjuncta*	Bjerke WP286-2 (TLE)	Norway	Tromso	KP771829
Montanelia	*disjuncta*	Wedin 7143 (UPS)	Sweden	Lycksele Lappmark	KP771834
Montanelia	*disjuncta*	Coppins s.n (MAF)	UK	Scotland	KP771835
Montanelia	*disjuncta*	MAF-Lich 15512	India	Uttaranchal	KP771837
Montanelia	*disjuncta*	Esslinger BP94-3 (TLE)	Canada	Yukon	KP771830
Montanelia	*disjuncta*	Esslinger BP94-3 (TLE)	Canada	Yukon	KF257967
Montanelia	*disjuncta*	LA28245	**Iceland**	Iau	**KY963377**
Montanelia	*disjuncta*	LA30617	**Iceland**	Ino	**KY508684**
Montanelia	*disjuncta*	LA27588	**Iceland**	Ino	**KY508685**
Montanelia	*disjuncta*	LA31552	**Iceland**	Ino	**KY963378**
Montanelia	*disjuncta*	LA30657	**Iceland**	Ino	**KY508686**
Montanelia	*panniformis*	Lumbsch 2010/M8 (F)	USA	Maine, Washington Co.	KF257973
Montanelia	*panniformis*	Harris 55589 (NY)	USA	Maine	KF257974
Montanelia	*panniformis*	Harris 54728 (NY)	USA	Maine	KF257975
Montanelia	*panniformis*	Harris 52834 (NY)	USA	Maine	KF257976
Montanelia	*panniformis*	Lumbsch 2010/M8	USA	Maine	KP771838
Montanelia	*panniformis*	Harris 55589	USA	Maine	KP771839
Montanelia	*panniformis*	Harris 54728 (NY)	USA	Maine	KP771840
Montanelia	*panniformis*	Harris 52834(NY)	USA	Maine	KP771841
Montanelia	*panniformis*	Ohmura 9654 (TNS)	Japan	Azusayama	KM386099
Montanelia	*sorediata*	Lendemer 13329 (NY)	USA	Pennsylvania	KF257978
Montanelia	*sorediata*	Lendemer 13329 (NY)	USA	Pennsylvania	KP771846
Montanelia	*sorediata*	Spribille 31972 (GZU)	Russia	Khabarovskiy Krai	KF257981
Montanelia	*sorediata*	Spribille 31972 (GZU)	Russia	Khabarovskiy Krai	KP771847
Montanelia	*sorediata*	Wedin 6862 (UPS)	Sweden	Vasterbotten	GU994557
Montanelia	*sorediata*	Wedin 6862 (UPS)	Sweden	Vasterbotten	KP771845
Montanelia	*sorediata*	Ohmura 9666 (TNS)	Japan	Mt. Ohyama	KM386101
Montanelia	*tominii*	s.n.	China	Heilongjiang	KF257983
Montanelia	*tominii*	s.n.	China	Heilongjiang	KF257984
Montanelia	*tominii*	Leavitt s.n. (F)	USA	Utah, Wayne Co.	KF257985
Montanelia	*tominii*	Esslinger 16627 (TLE)	USA	Montana	KF257986
Montanelia	*tominii*	32307 (BRY-C)	USA	New Mexico, Catron Co.	KF257987
Montanelia	*tominii*	Henson 54 (TLE)	USA	Colorado	KF257988
Montanelia	*tominii*	Leavitt 1021	USA	Utah, Wayne Co.	KF257989
Montanelia	*tominii*	Leavitt 1022	USA	Utah, Wayne Co.	KF257990
Montanelia	*tominii*	Lendemer 4494 (NY)	USA	California	KF257991
Montanelia	*tominii*	Lendemer 19701 (NY)	USA	California	KF257992
Montanelia	*tominii*	Esslinger BP20-1 (TLE)	Canada	Yukon Territory	KF257998
Montanelia	*tominii*	HMAS-L-114000	China	Hebei, Mt. Wulingshan	EU784154
Montanelia	*tominii*	HMAS-L-036389	China	Inner Mongolia, Mt. Arxan	EU784155
Montanelia	*tominii*	HMAS-L-071058	China	Inner Mongolia, Bairin Youqi	EU784156
Montanelia	*tominii*	17025 (MAF-Lich)	India	Sikkim	KF257999
Montanelia	*tominii*	15516 (MAF-Lich)	India	Uttaranchal	GU994559
Montanelia	*tominii*	Hutten 14162 (YOSE)	USA	California	KP771848
Montanelia	*tominii*	Esslinger 16627 (TLE)	USA	Montana	KP771849
Montanelia	*tominii*	Lendemer 4494 (NY)	USA	California	KP771854
Montanelia	*tominii*	Lendemer 19701 (NY)	USA	California	KP771855
Montanelia	*tominii*	Henson 54 (TLE)	USA	Colorado	KP771851
Montanelia	*tominii*	Leavitt 1022 (F)	USA	Utah	KP771853
Montanelia	*tominii*	32307 (BRY-C)	USA	New Mexico, Catron Co.	KP771850
Montanelia	*tominii*	Leavitt 1021 (F)	USA	Utah	KP771852
Montanelia	*tominii*	Ren, Qiang s.n. (HMAS-L)	China	Heilongjiang	KP771856
Montanelia	*tominii*	Ren, Qiang s.n. (HMAS-L)	China	Heilongjiang	KP771858
Montanelia	*tominii*	Esslinger BP20-1 (TLE)	Canada	Yukon	KP771857
