# Supplementary material for: DNA barcoding and LC-MS metabolite profiling of the lichen-forming genus Melanelia: Specimen identification and discrimination focusing on Icelandic taxa
Source: PLoS One. 2017 May 24;12(5):e0178012. doi: 10.1371/journal.pone.0178012 (PMC5443556; doi:10.1371/journal.pone.0178012)
Supplement: S1 Fig — (A) cryptostictic acid 1. (B) stictic acid 2. (C) norstictic acid 3. (PDF) [file pone.0178012.s002.pdf]

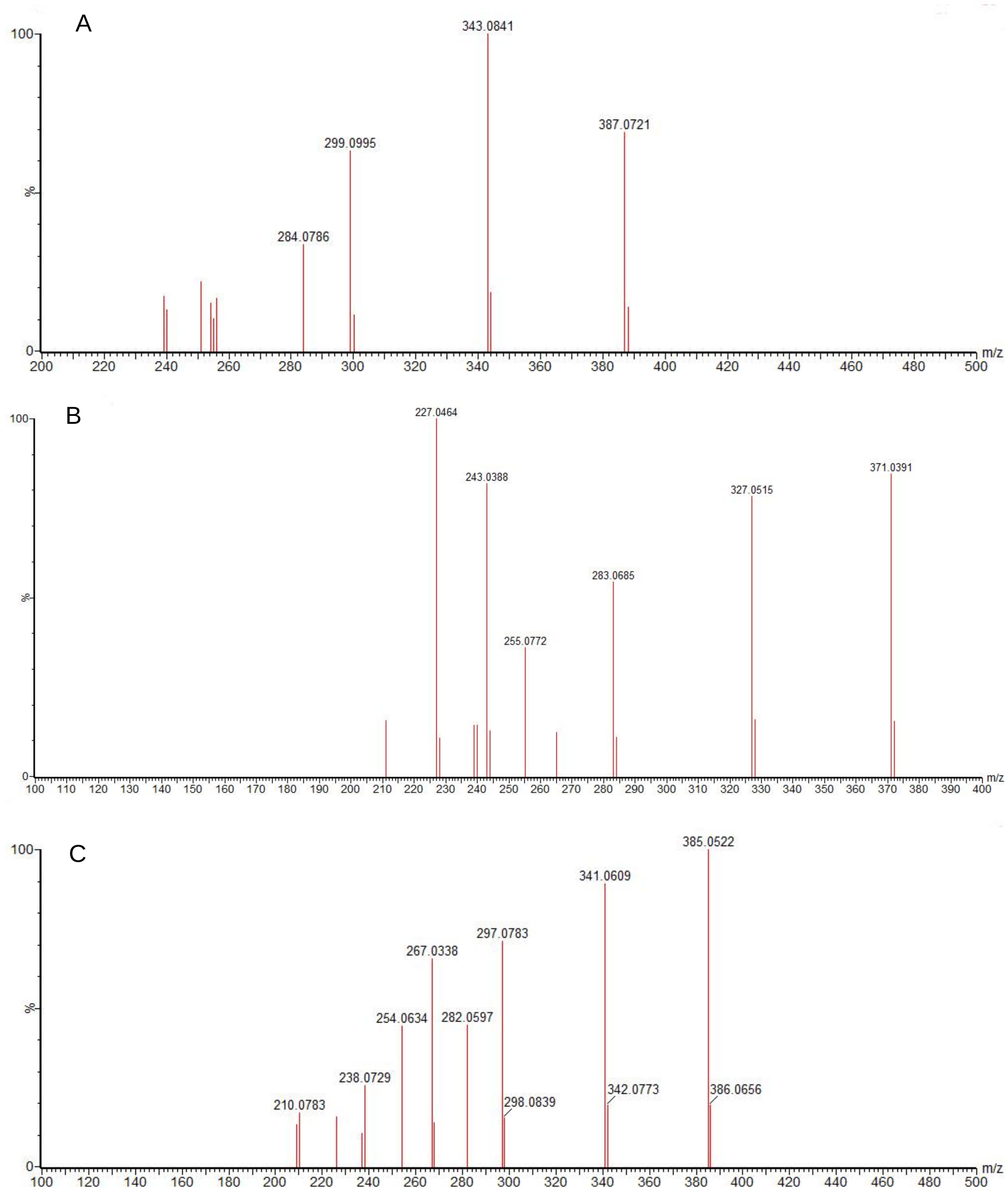

S1 Fig. MS^2 spectra of depsidones in the lichen *Melanelia hepatizon*. (A) cryptostictic acid **1**. (B) stictic acid **2**. (C) norstictic acid **3**.
