# Supplementary material for: DNA barcoding and LC-MS metabolite profiling of the lichen-forming genus Melanelia: Specimen identification and discrimination focusing on Icelandic taxa
Source: PLoS One. 2017 May 24;12(5):e0178012. doi: 10.1371/journal.pone.0178012 (PMC5443556; doi:10.1371/journal.pone.0178012)
Supplement: S2 Fig — (A) stenosporic acid 5. (B) perlatolic acid 7. (PDF) [file pone.0178012.s003.pdf]

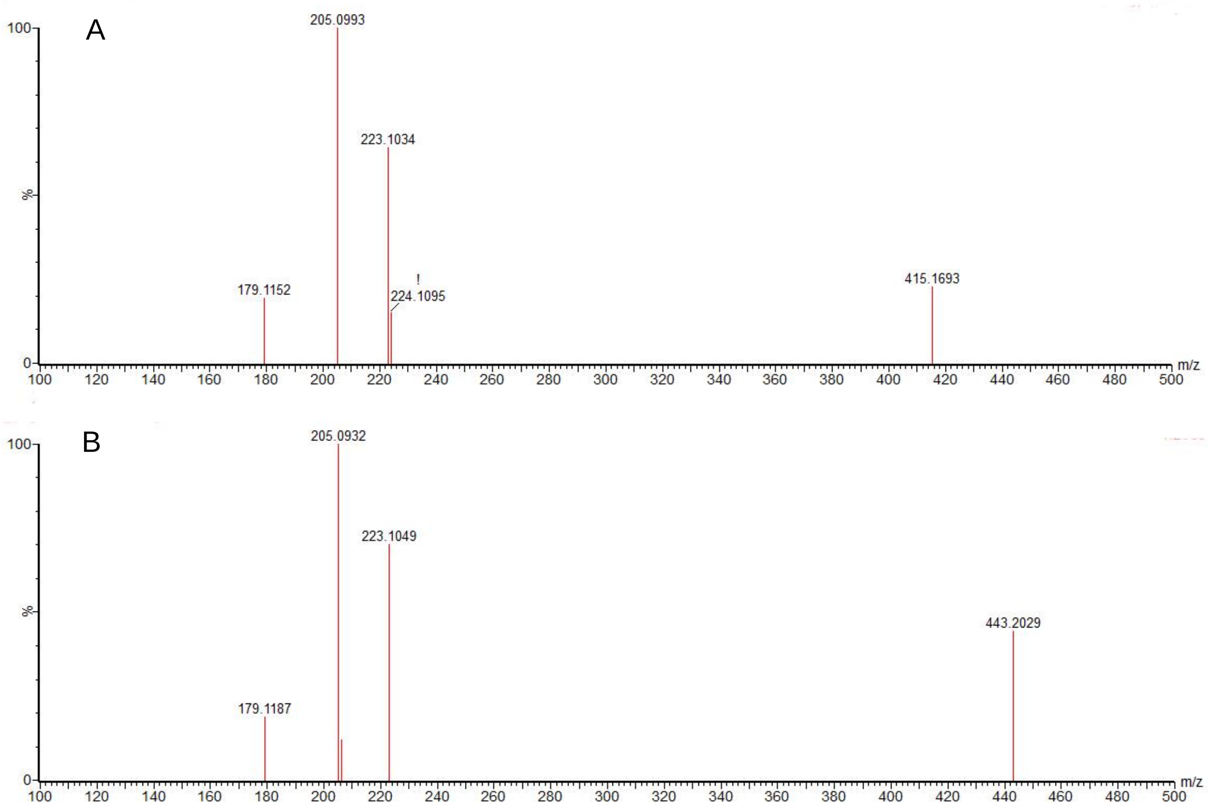

S2 Fig. MS^2 spectra of depsides in the lichen *Montanelia disjuncta*. (A) stenosporic acid **5**. (B) perlatolic acid **7**.
