# Supplementary material for: DNA barcoding and LC-MS metabolite profiling of the lichen-forming genus Melanelia: Specimen identification and discrimination focusing on Icelandic taxa
Source: PLoS One. 2017 May 24;12(5):e0178012. doi: 10.1371/journal.pone.0178012 (PMC5443556; doi:10.1371/journal.pone.0178012)
Supplement: S3 Fig — (A) MS spectrum of usnic acid 4 showing the adduct ion at m/z 709.1456 and molecular ion at m/z 343.0847. (B) MS2 spectrum of the usnic acid molecular ion. (PDF) [file pone.0178012.s004.pdf]

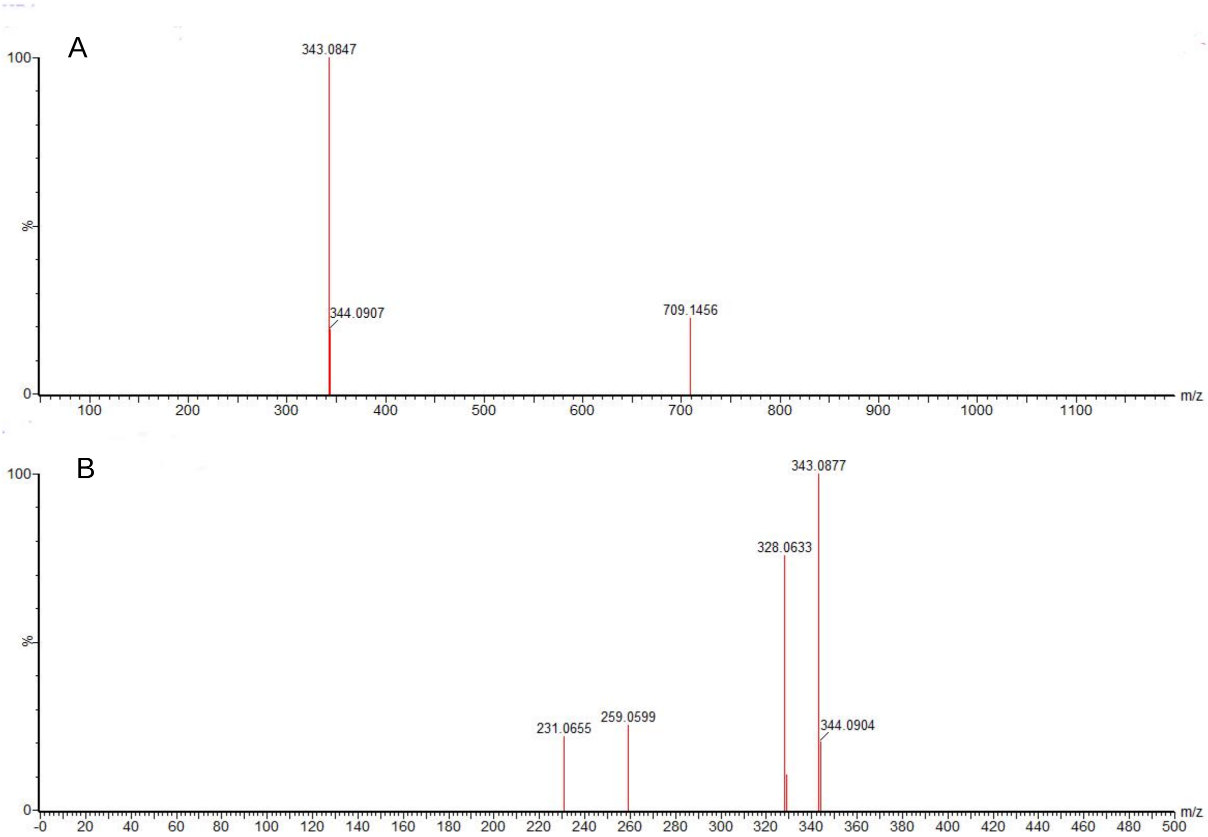

S3 Fig. MS and MS^2 spectra of usnic acid in one chemotype of the lichen *Montanelia disjuncta*.
(A) MS spectrum of usnic acid **4** showing the adduct ion at m/z 709.1456 and molecular ion at m/z 343.0847. (B) MS^2 spectrum of the usnic acid molecular ion.
