# Supplementary material for: DNA barcoding and LC-MS metabolite profiling of the lichen-forming genus Melanelia: Specimen identification and discrimination focusing on Icelandic taxa
Source: PLoS One. 2017 May 24;12(5):e0178012. doi: 10.1371/journal.pone.0178012 (PMC5443556; doi:10.1371/journal.pone.0178012)

S6 Fig. Baysian nrITS gene tree from 116 specimens representing all *Melanelia* and *Montanelia* species.
Posterior probabilities over 0.94 are labelled above branches.

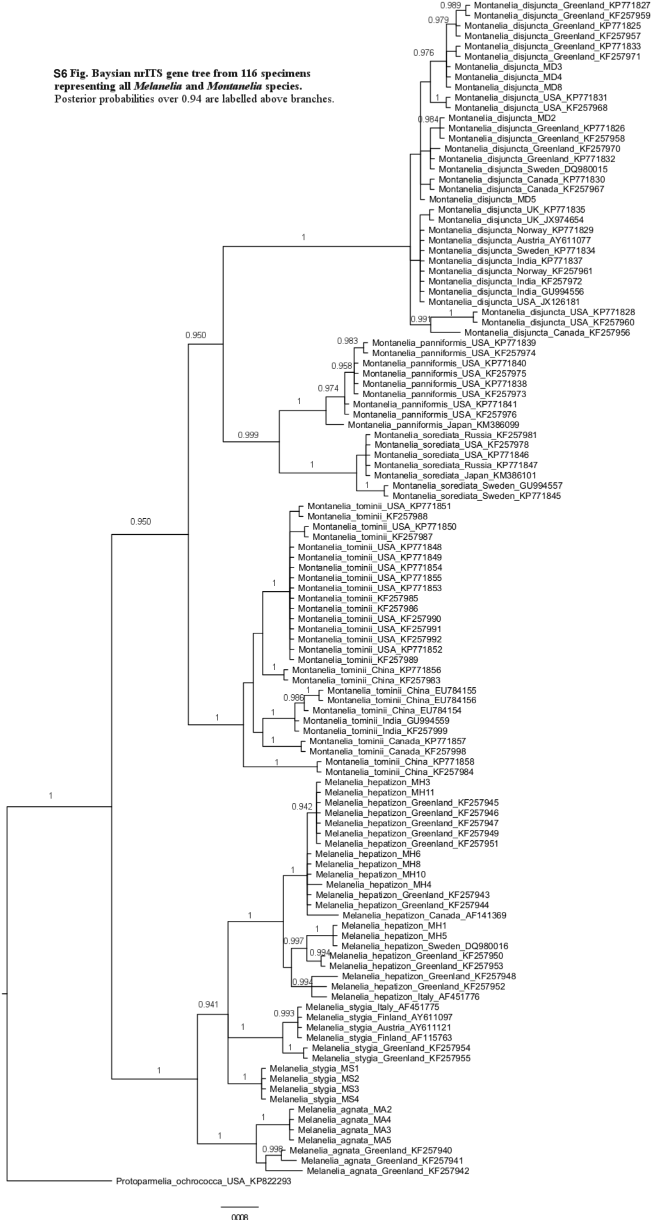

Supplement: S6 Fig — Posterior probabilities over 0.94 are labelled above branches. (PDF) [file pone.0178012.s007.pdf]
