# Supplementary material for: DNA barcoding and LC-MS metabolite profiling of the lichen-forming genus Melanelia: Specimen identification and discrimination focusing on Icelandic taxa
Source: PLoS One. 2017 May 24;12(5):e0178012. doi: 10.1371/journal.pone.0178012 (PMC5443556; doi:10.1371/journal.pone.0178012)
Supplement: S7 Fig — Bootstrap values (%) over 70 are labelled above branches. (PDF) [file pone.0178012.s008.pdf]

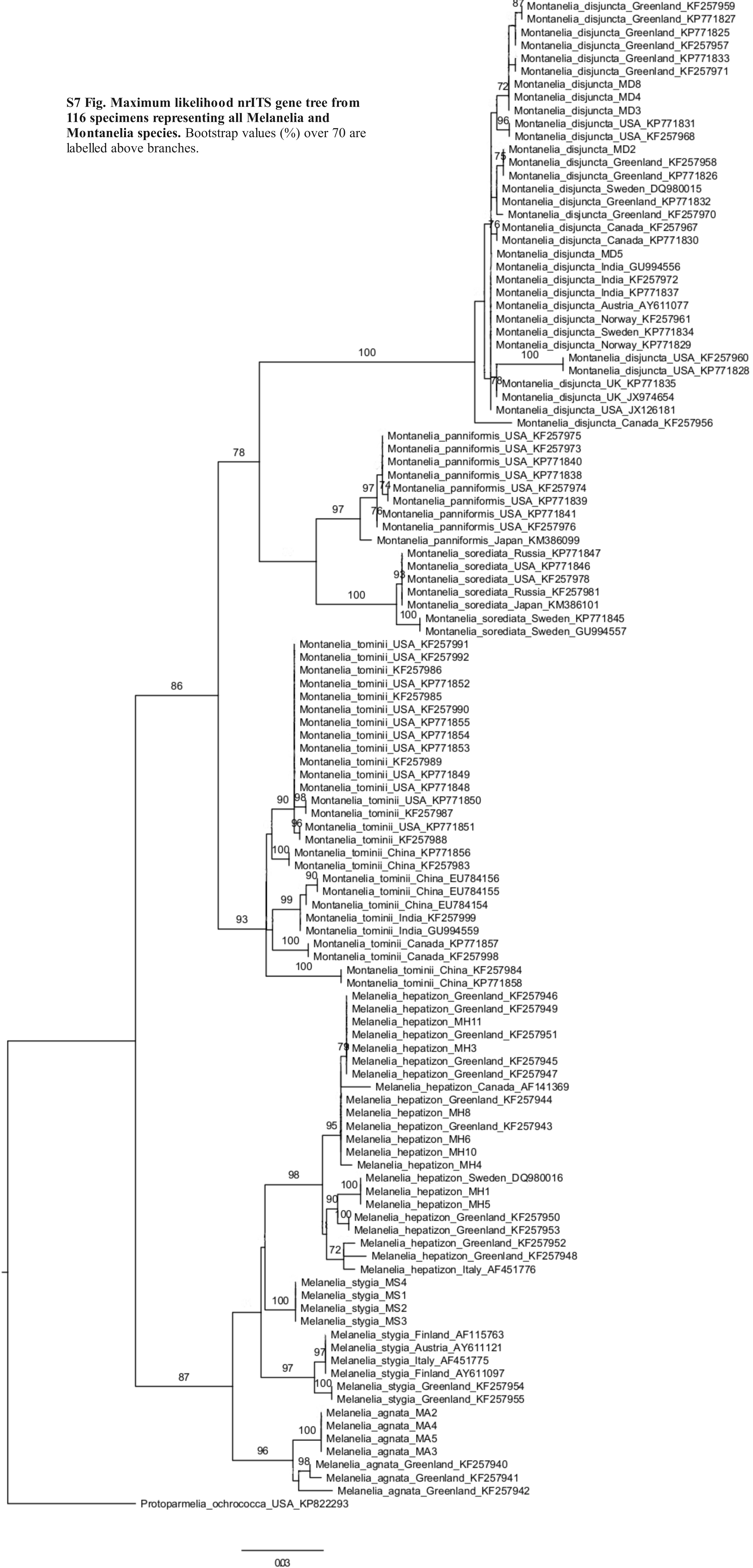

S7 Fig. Maximum likelihood nrITS gene tree from 116 specimens representing all Melanelia and Montanelia species. Bootstrap values (%) over 70 are labelled above branches.
