# Supplementary material for: DNA barcoding and LC-MS metabolite profiling of the lichen-forming genus Melanelia: Specimen identification and discrimination focusing on Icelandic taxa
Source: PLoS One. 2017 May 24;12(5):e0178012. doi: 10.1371/journal.pone.0178012 (PMC5443556; doi:10.1371/journal.pone.0178012)
Supplement: S4 Fig — (PDF) [file pone.0178012.s005.pdf]

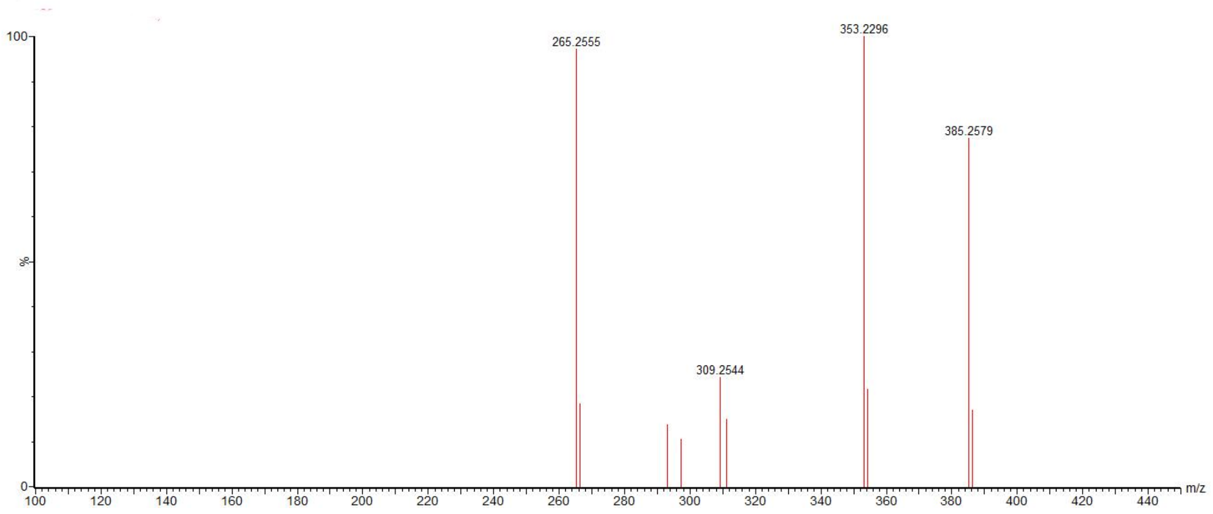

S4 Fig. MS^2 spectrum of rangiformic acid.
